# Selective targeting of mRNA and the following protein synthesis of CaMKIIα at the long-term potentiation-induced site

**DOI:** 10.1242/bio.042861

**Published:** 2020-01-23

**Authors:** Itsuko Nihonmatsu, Noriaki Ohkawa, Yoshito Saitoh, Reiko Okubo-Suzuki, Kaoru Inokuchi

**Affiliations:** 1 Mitsubishi Kagaku Institute of Life Sciences, MITILS, 11 Minamiooya, Machida, Tokyo 194-8511, Japan; 2 Division for Memory and Cognitive Function, Research Center for Advanced Medical Science, Comprehensive Research Facilities for Advanced Medical Science, Dokkyo Medical University, 880 Kita-kobayashi, Mibu-machi, Shimotsuga-gun, Tochigi 321-0293, Japan; 3 Department of Biochemistry, University of Toyama Graduate School of Medicine and Pharmaceutical Sciences, 2630 Sugitani, Toyama 930-0194, Japan; 4 PRESTO, Japan Science and Technology Agency (JST), 4-1-8 Honcho, Kawaguchi, Saitama 332-0012, Japan; 5 CREST, JST, University of Toyama, 2630 Sugitani, Toyama 930-0194, Japan

**Keywords:** CaMKIIα, *Camk2a* mRNA, Dentate gyrus, Hippocampus, Local protein synthesis, Long-term potentiation

## Abstract

Late-phase long-term potentiation (L-LTP) in hippocampus, thought to be the cellular basis of long-term memory, requires new protein synthesis. Neural activity enhances local protein synthesis in dendrites, which in turn mediates long-lasting synaptic plasticity. Ca^2+^/calmodulin-dependent protein kinase IIα (CaMKIIα) is a locally synthesized protein crucial for this plasticity, as L-LTP is impaired when its local synthesis is eliminated. However, the distribution of *Camk2a* mRNA during L-LTP induction remains unclear. In this study, we investigated the dendritic targeting of *Camk2a* mRNA after high-frequency stimulation, which induces L-LTP in synapses of perforant path and granule cells in the dentate gyrus *in vivo*. *In situ* hybridization studies revealed that *Camk2a* mRNA was immediately but transiently targeted to the site receiving high-frequency stimulation. This was associated with an increase in *de novo* protein synthesis of CaMKIIα. These results suggest that dendritic translation of CaMKIIα is locally mediated where L-LTP is induced. This phenomenon may be one of the essential processes for memory establishment.

## Abstract (precis)

**Summary:** Selective targeting of mRNA and the following protein synthesis of CaMKIIalpha at neuronal plasticity-induced sites may be one of important processes for establishment of long-term memory.

## INTRODUCTION

Macromolecular synthesis induced by neural activity is essential for the neural plasticity that underlies memory formation, such as late-phase long-term potentiation (L-LTP) in the hippocampus (Frey and Morris, 1997; Nakahata and Yasuda, 2018; Nguyen and Kandel, 1997). A model of synaptic tagging involving cell-wide molecular events may explain late-phase plasticity at activated postsynaptic sites (Frey and Morris, 1997; Okada and Inokuchi, 2015; Okada et al., 2009). Another model involves the local synthesis of proteins, because protein synthesis inhibitors applied to the dendritic fields impair L-LTP (Bradshaw et al., 2003; Sutton and Schuman, 2006). The discovery of polyribosomes at the base of dendritic spines (Steward and Levy, 1982) suggests that protein synthesis might be regulated at synapses. Moreover, dendritic RNAs are redistributed by neural activity (Matsumoto et al., 2007) and may be targeted to activated synaptic sites for local protein synthesis. For example, newly synthesized *Arc* mRNA is selectively localized near activated synaptic sites in response to neural activation (Steward et al., 1998).

The mRNA for the α subunit of Ca^2+^/calmodulin-dependent protein kinase II (CaMKIIα) is also found in dendritic shafts (Burgin et al., 1990; Miller et al., 2002). CaMKII is a multifunctional serine/threonine kinase that participates in the Ca^2+^-sensitive processes underlying the short- and long-term regulation of synapses and memory (Lisman et al., 2002, 2012). Hippocampal L-LTP is suppressed when the translocation of *Camk2a* mRNA to dendrites is blocked (Miller et al., 2002), indicating that this targeting is important for neural plasticity. In addition, the induction of LTP at perforant path (PP)–granule cell synapses in the dentate gyrus enhances the expression of *Camk2a* mRNA in synaptodendrosomes (Håvik et al., 2003). However, whether this induction also mediates the selective targeting and translation of *Camk2a* mRNA at activated sites is not clear.

In the dentate gyrus, the lateral PP, the medial PP, and the major portion of the hilar projection to the molecular layer (ML) comprise the outer (OML), middle (MML) and inner (IML) molecular layers, respectively (Steward, 1976; Tamamaki, 1999). Synaptic activation increases the immunoreactivity for CaMKIIα, specifically in the activated lamina of the ML (Steward and Halpain, 1999). The increase can be detected after 5 min of stimulation and becomes more distinct with longer stimulation (∼2 h) (Steward and Halpain, 1999). The authors of that study also reported that the increase was not sensitive to inhibitors of protein synthesis (Steward and Halpain, 1999), and the origin of the increased CaMKIIα was not clear. The selective distribution of *Camk2a* mRNA in activated lamina has not been observed after longer layer-specific activation (Steward et al., 1998). Nevertheless, a re-evaluation of mRNA distribution under conditions for L-LTP induction may provide meaningful insight into the underlying physiological mechanisms.

In this study, we found that the induction of L-LTP in the dentate gyrus regions of freely moving rats rapidly increased *Camk2a* mRNA and protein in the corresponding ML where granule cell dendrites extend. Furthermore, this increase correlated with the accumulation of actin filaments (F-actin), which we previously showed are involved in L-LTP induction and maintenance (Fukazawa et al., 2003; Nihonmatsu et al., 2015; Ohkawa et al., 2012). The targeting of *Camk2a* mRNA to activated sites was transient, and the corresponding increase in protein was protein synthesis-dependent, suggesting that the targeted *Camk2a* mRNA was locally synthesized, a phenomenon that may be important for transitioning from the early to late phase of LTP.

## RESULTS

### F-actin rapidly and persistently increases in MML after L-LTP induction

**Fig. 1.**
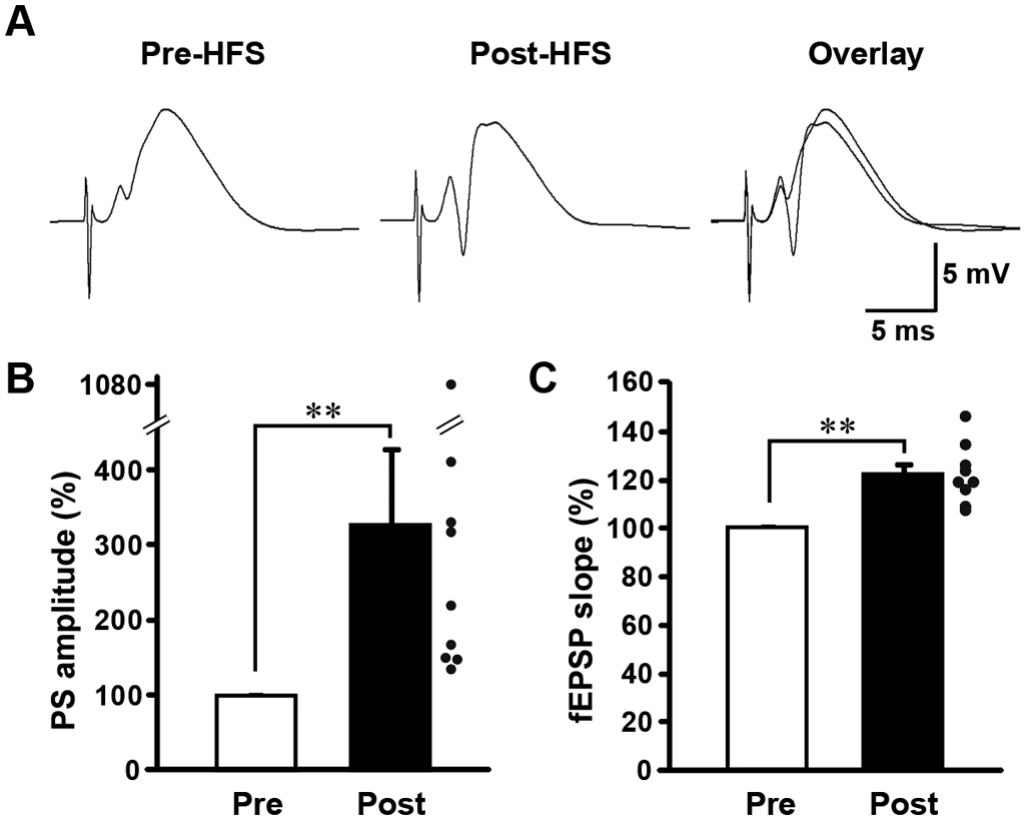
**HFS induces L-LTP in dentate gyrus of freely moving animals.** (A) Typical wave forms pre- and post-HFS of the PP. Average (for 15 min) PS amplitudes (B) and fEPSP slopes (C) pre- and post-HFS. Error bars indicate mean±s.e.m (*n*=9). **P<0.01 by Wilcoxon signed-rank test (B) and paired *t*-test (C).

**Fig. 2.**
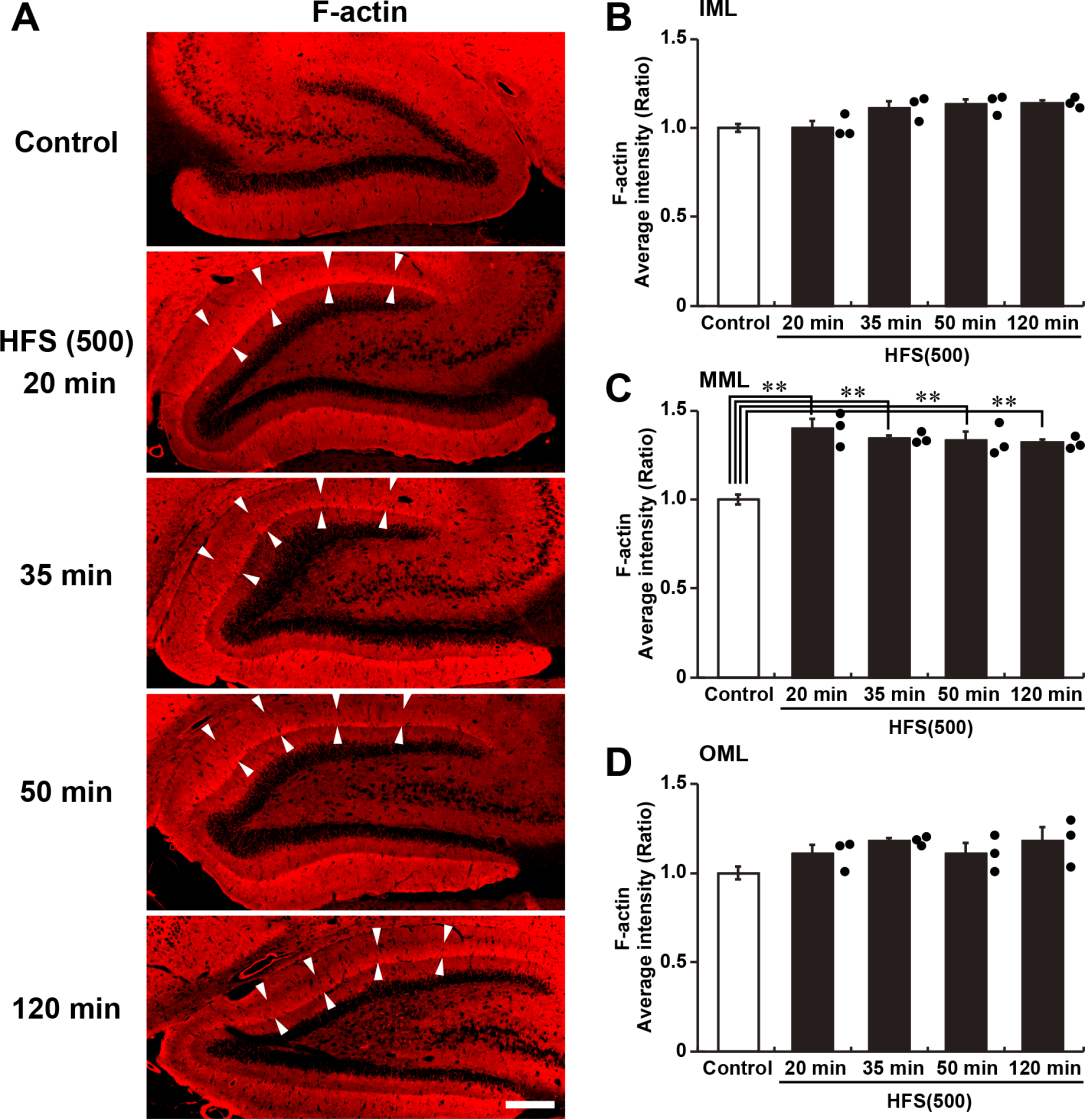
**F-actin levels in the dentate gyrus after HFS.** (A) Micrographs of F-actin by phalloidin-rhodamine staining in dentate gyrus. Arrowheads indicate the MML of upper blade where HFS was delivered. Scale bar: 300 µm. (B–D) Average intensities of F-actin staining in the IML (B), MML (C) and OML (D). Graphs show relative indices compared with control dentate gyrus. Control, *n*=10; HFS(500), *n*=3 at each time point. Error bars indicate mean±s.e.m. (B) *P*<0.01 by one-way ANOVA; (C) *P*<0.001 by one-way ANOVA, **P<0.01 according to Scheffé’s *post hoc* test; (D) *P*>0.05 by one-way ANOVA.

**Fig. 3.**
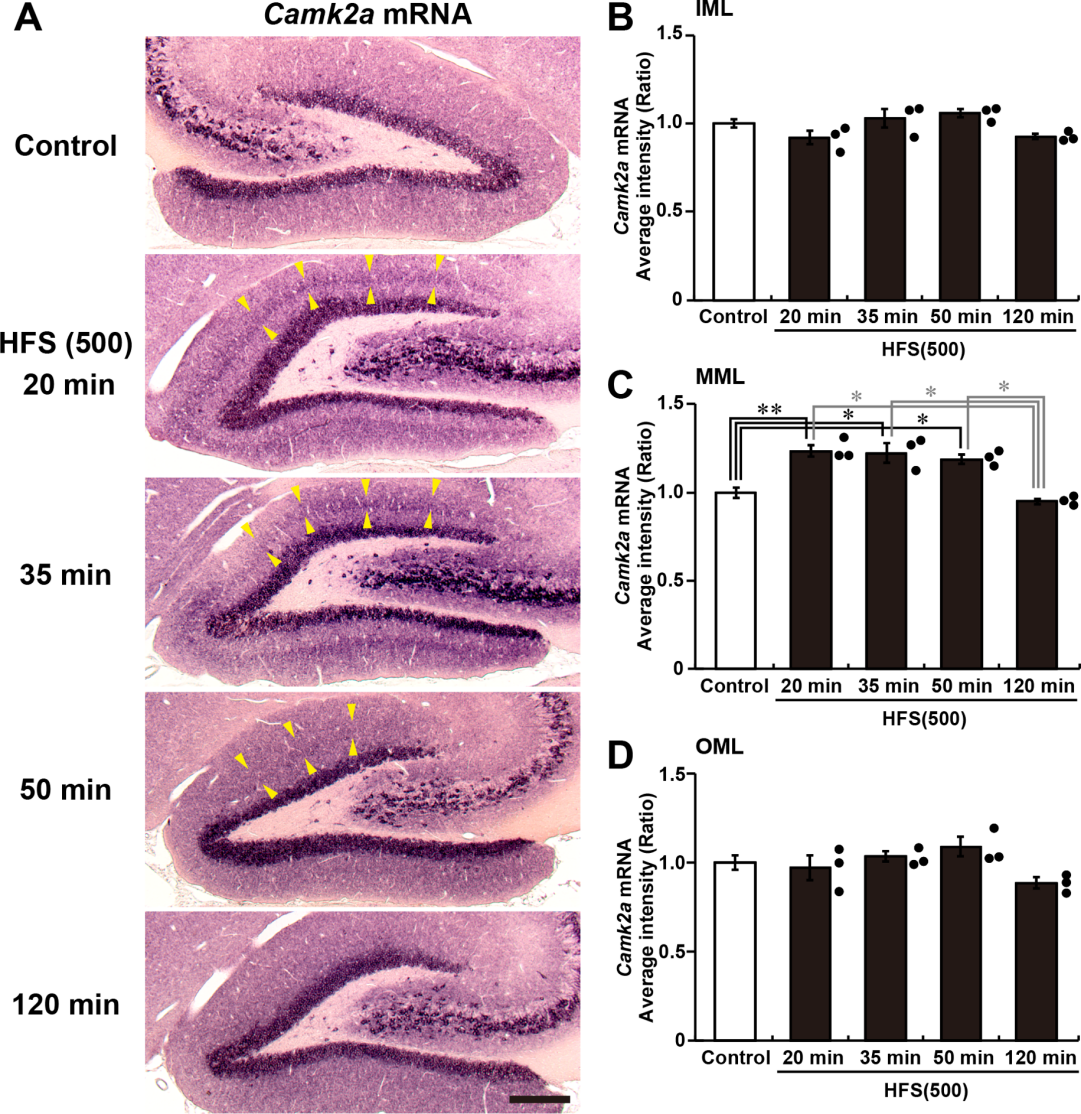
***Camk2a* mRNA is transiently targeted to sites of L-LTP induction.** (A) Micrographs of *Camk2a* mRNA observed by *in situ* hybridization in dentate gyrus. Arrowheads indicate the MML of upper blade where HFS was delivered. Scale bar: 300 µm. (B–D) Average intensities of *Camk2a* mRNA in the IML (B), MML (C) and OML (D). Graphs show relative indices of average signal intensity compared with control dentate gyrus. Control, *n*=10; HFS(500), *n*=3, each time point. Error bars indicate mean±s.e.m. (B) *P*>0.11 by one-way ANOVA; (C) *P*<0.001 by one-way ANOVA, *P<0.05, **P<0.01 according to Scheffé’s *post hoc* test; (D) *P*>0.23 by one-way ANOVA.

High-frequency stimulation (HFS) was delivered to PP fibers in freely moving adult rats, which induces a potentiation of the population spike (PS) amplitude that persists for at least 7 days (Fukazawa et al., 2003; Ohkawa et al., 2012). Accordingly, PS amplitudes (327.82±100.06) and field excitatory postsynaptic potential (fEPSP) slopes (122.21%±4.08%) were increased 15 min after HFS (Fig. 1) was applied to all nine animals investigated in Figs 2 and 3, except for an ‘HFS(500) 20 min’ condition.

Our previous work also demonstrated that the induction of L-LTP in the dentate gyrus *in vivo* reorganizes the actin cytoskeleton, observed as an increase in F-actin that persists for several weeks (Fukazawa et al., 2003; Ohkawa et al., 2012). Accordingly, an increase in F-actin in the ML was detected by phalloidin staining as early as 20 min after HFS was applied and persisted for at least 120 min (Fig. 2A). The increase was significant only in the MML, with no difference in the average intensities in the IML (nearest the somas) or in the OML (distal from the somas) between control and HFS conditions (Fig. 2B–D). These data strongly suggest that L-LTP induction induces actin reorganization specifically in the MML of the upper blade of the dentate gyrus.

### *Camk2a* mRNA is targeted to dendrites after L-LTP induction

Dendritic translocation of *Camk2a* mRNA is required for hippocampal L-LTP (Miller et al., 2002). To determine whether the induction of L-LTP targets these transcripts to activated sites, we performed *in situ* hybridization on sections containing the dentate gyrus from rats after HFS was applied. We focused the ML of upper blade because F-actin accumulation is induced at only the MML but not at the IML and OML of upper blade in the all animals analyzed (Fig. 2). Quantitative analyses revealed an increase in *Camk2a* mRNA in the MML beginning 20 min after HFS was applied (Fig. 3A,C). Consistent with the phalloidin staining, there was no increase in the IML or OML (Fig. 3B,D). However, at 120 min after HFS was applied, the levels of *Camk2a* mRNA in the MML returned to control levels. These results indicate that *Camk2a* mRNA was immediately but transiently targeted to the MML where LTP was induced.

### *De novo* synthesis of CaMKIIα is selectively increased in dendrites after L-LTP induction

**Fig. 4.**
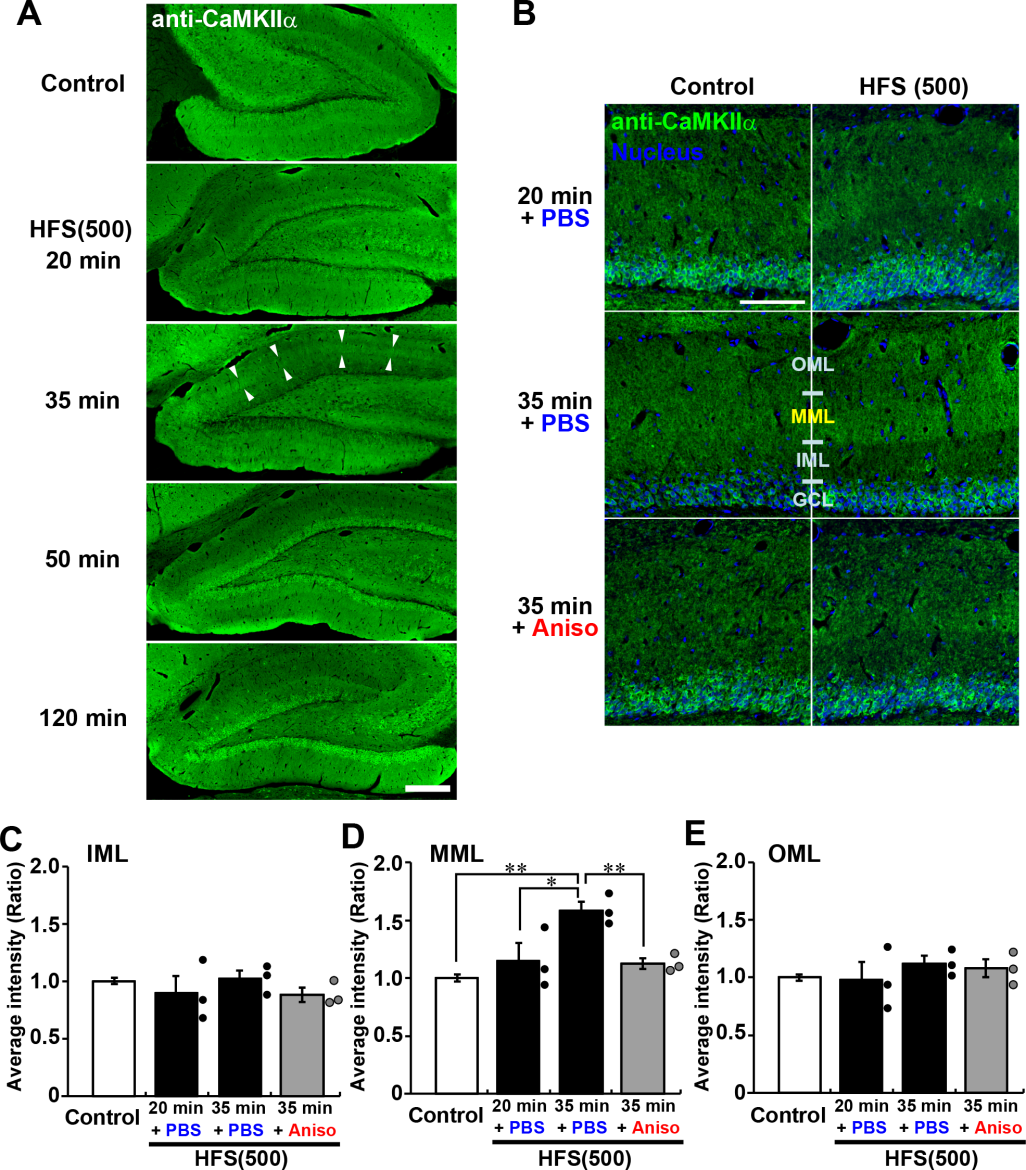
***De novo* synthesis of CaMKIIα protein after the HFS delivery.** (A) Micrographs of immunoreactivity of CaMKIIα protein observed in contralateral (control) and stimulated dentate gyrus. Arrowheads indicate MML where HFS was delivered. Scale bar: 300 µm. (B) Micrographs of immunoreactivity for CaMKIIα protein under conditions of control and HFS delivery with PBS or anisomycin (Aniso) infusion. Scale bar: 100 µm. (C–E) Average intensities of CaMKIIα immunoreactivity in the IML (C), MML (D) and OML (E). Graphs show relative indices of average signal intensity compared with control dentate gyrus. Control, *n*=9; HFS(500), *n*=3, each condition. (C) *P*>0.41 by one-way ANOVA; (D) *P*<0.001 by one-way ANOVA, *P<0.05, **P<0.01 according to Scheffé’s *post hoc* test; (E) *P*>0.50 by one-way ANOVA.

To determine whether the targeted *Camk2a* mRNA mediates local translation, we performed immunohistochemistry for CaMKIIα in the dentate gyrus following HFS. While an increase in mRNA peaked at 20–35 min after HFS, an increase in immunoreactivity for CaMKIIα was detected in the MML 35 min after HFS was applied (Fig. 4A). To verify that the increase was a result of *de novo* synthesis, anisomycin was infused into the lateral ventricles immediately following LTP induction. HFS no longer resulted in increased CaMKIIα in the MML when the protein synthesis inhibitor was applied (Fig. 4B,D). Consistent with the F-actin and mRNA results, these effects were only observed in the MML. The spatio-temporal expression pattern of the mRNA and protein strongly suggests that the *Camk2a* mRNA targeted to the MML after L-LTP induction was locally translated.

## DISCUSSION

*Camk2a* mRNA is abundantly and constitutively expressed in dendrites of dentate granule cells (Burgin et al., 1990; Paradies and Steward, 1997; Sutton and Schuman, 2006), and *Camk2a* mutant mice exhibit impaired hippocampal LTP and hippocampus-dependent learning (Giese et al., 1998; Lisman et al., 2002, 2012; Silva et al., 1992a,b). Furthermore, dendritic translocation of *Camk2a* mRNA is important for L-LTP but not early-phase LTP in the hippocampus (Miller et al., 2002). Thus, local synthesis of CaMKIIα in hippocampal dendrites may be required for the establishment of L-LTP and hippocampus-dependent memory formation. Here, we demonstrate that HFS of the hippocampus *in vivo* results in the rapid (within 20 min) but transient targeting of *Camk2a* mRNA and *de novo* synthesis of CaMKIIα in activated dendritic regions of the dentate gyrus. This stimulation can induce potentiation that persists for at least 1 week (Fukazawa et al., 2003), which corresponds to L-LTP establishment. Similar protocols trigger rapid and transient delivery of pre-existing *Camk2a* mRNA to synaptodendrosomes containing pinched-off dendritic spines (Håvik et al., 2003). Together, these data indicate that *Camk2a* mRNA is translocated selectively to activated dendritic spines immediately after LTP induction before returning to baseline levels after approximately 120 min.

We observed a selective and transient increase in CaMKIIα in the MML after *in vivo* HFS of the PP, consistent with the increased protein levels in synaptodendrosome fractions reported previously (Håvik et al., 2003). We found that the increase was restricted to the MML, corresponding to the increases in F-actin and mRNA in the MML but not the IML or OML. Notably, the increase in protein was detected 35 min after HFS was applied and was blocked by infusion of anisomycin. These data strongly indicate that the transiently targeted *Camk2a* mRNA is locally synthesized in dendritic regions where LTP is induced.

HFS induces localized phosphorylation of ribosomal protein S6, a component of the 40S ribosome detected in polyribosome-enriched fractions from cultured cortical neurons (Krichevsky and Kosik, 2001) and associated with initiating the translation of certain mRNAs (Pirbhoy et al., 2016, 2017). Electron microscopy has revealed that the S6 immunoreactivity in dendritic spines is transiently increased at sites were LTP is induced (Nihonmatsu et al., 2015), with a time course similar to that for *Camk2a* mRNA. Moreover, depolarization of synaptosomal membranes results in an increased association between polysomes and *Camk2a* mRNA and increased synthesis of CaMKIIα protein (Bagni et al., 2000). There is evidence supporting the idea that mRNA is rapidly transported into activated spines with polysomes and translated during L-LTP establishment. Nevertheless, CaMKIIα expression in the ML induced by prolonged HFS (∼2 h) was reported to be independent of protein synthesis, although inhibitors diminished (∼25%) the immunoreactivity (Steward and Halpain, 1999). The data we present here support that local translation of transiently targeted *Camk2a* mRNA contributes in part to the increase in CaMKIIα at activated synaptic sites during the establishment of L-LTP *in vivo*.

Neuronal inputs that correspond with induction of different forms of neuronal plasticity attract various mRNAs and their binding proteins to activated sites in a stimulation pattern-dependent manner (Leal et al., 2014; Tiruchinapalli et al., 2003; Yoon et al., 2016). Selective targeting of mRNA followed by local translation is strictly regulated by combination between input pattern and cis-element (Wang et al., 2009). Difference of activation patterns between the present and a previous study (Steward and Halpain, 1999) may be the reason why targeting of *Camk2a* mRNA in the activated layer of DG was observed or not. In addition to translocation, selective degradation of mRNAs should be considered for input-specific targeting of mRNAs on activated synapses. In *Drosophila*, local translation of *Camk2* mRNA is controlled by a balance between RISC, a component of RNA interference, and proteasome, which works for degradation of RISC (Ashraf et al., 2006). These accurate regulations of local translation probably increase signal and noise ratio of synapses to establish circuits for cognitive functions, on the other hand, disruption of the system may link to cognitive impairments and neuropsychiatric disorders (Khlebodarova et al., 2018).

## MATERIALS AND METHODS

### Animals

These studies were performed using male Wistar ST rats (Sankyo Labo Service Corporation, Inc., Tokyo, Japan) approximately 20 weeks of age. All procedures involving the use of animals complied with the guidelines of the National Institutes of Health and were approved by the Animal Care and Use Committee of Mitsubishi Kagaku Institute of Life Sciences and the University of Toyama.

### Dentate gyrus LTP in un-anaesthetized freely moving animals

The surgical procedure to induce LTP was as described previously (Fukazawa et al., 2003; Kato et al., 1998, 1997; Ohkawa et al., 2012; Okubo-Suzuki et al., 2016). The electrode-stimulating PP fibers were positioned 8.0 mm posterior, 4.5 mm lateral and 5.0 mm inferior to bregma. A recording electrode was implanted ipsilaterally 4.0 mm posterior, 2.5 mm lateral and 3.8 mm ventral to bregma. For intraventricular infusions, a stainless-steel guide cannula (Eicom, Kyoto, Japan) was positioned ipsilateral at 0.8–1.0 mm posterior, 1.6 mm lateral and 4.0 mm ventral to bregma. After surgery, a dummy cannula (Eicom), which extended 1.0 mm beyond the end of the guide cannula, was inserted into the guide cannula, as in our previous report (Okubo-Suzuki et al., 2016).

LTP experiments on freely moving animals were performed as described previously (Fukazawa et al., 2003; Matsuo et al., 2000; Ohkawa et al., 2012). LTP was induced by tetanic stimulation comprising biphasic square waveforms at a pulse width of 200 µs. The intensity of the stimulus current was set to elicit 60% of the maximal PS amplitude. The animals were placed in the recording chamber, and the baseline response was monitored by delivering test pulses (0.05 Hz) for 15 min (Fig. 1). LTP was then induced using 500 pulses of HFS consisting of 10 trains with 1 min intertrain intervals (total, 10 min). Each train consisted of five bursts of 10 pulses at 400 Hz, delivered at 1 s interburst intervals. Synaptic transmission was monitored for 15 min after the delivery of HFS (Fig. 1), and then the rats were immediately given intraventricular infusions of a protein synthesis inhibitor. For this, the dummy cannula was removed, and an injection cannula (Eicom), which extends 0.5 mm beyond the end of the guide cannula, was inserted into each of the unanesthetized rats. Anisomycin (Sigma-Aldrich) dissolved in HCl was diluted with phosphate-buffered saline (PBS), and the pH was adjusted to 7.4 with NaOH. The anisomycin (600 µg/5 µl) or PBS as a control was infused slowly into the lateral ventricles of the freely moving rats via an infusion pump at a rate of 1 µl/min, as in our previous report (Okubo-Suzuki et al., 2016).

### *In situ* hybridization

A cDNA fragment comprising the 3′ untranslated region of *Camk2a* (nucleotides 1–548; GenBank accession number AB056125) from rat brain was obtained by reverse transcription-PCR and subcloned into the vector pCRII-TOPO. The vector was digested at each end of the *Camk2a* cDNA sequence to generate a template for *in vitro* transcription to produce an antisense or sense cRNA probe. Digoxigenin-labeled cRNA probes were produced by transcription with T7 or Sp6 RNA polymerase (Roche Diagnostics, Somerville, NJ).

*In situ* hybridization using the cRNA probes was performed as previously described (Hatanaka and Jones, 1998). Briefly, rats were deeply anesthetized with sodium pentobarbital (60 mg/kg body weight, intraperitoneal) and perfused with PBS and then with 4% paraformaldehyde and 0.05% glutaraldehyde in PBS (pH 7.4). The brains were removed and equilibrated in 25% sucrose in PBS for sectioning. Mounted sections (10 µm thickness) were fixed in 4% paraformaldehyde in PBS and permeabilized in Triton X-100 before treatments with HCl to hydrolyze nucleic acids and proteinase K to digest proteins. The sections were prehybridized with 2× SSC containing 50% formamide at 65°C and then hybridized with the digoxigenin-labeled cRNA probes in 5× SSC, 2% blocking reagent and 50% formamide at 60°C overnight. For the control conditions, the probe was omitted from the hybridization buffer and the antisense probe was replaced with a poly(dA) sense probe. The sections were then washed in 5× SSC containing 50% formamide at 65°C, treated with RNaseA, and rinsed in buffer. The probes were then detected using an anti-digoxigenin antibody coupled to alkaline phosphatase (Roche Diagnostics) according to the manufacturer's instructions; the enzymatic reaction was stopped in 10 µM Tris-HCl, 1 mM EDTA and then sections were mounted. Images were obtained with a light microscope (AX-80T; Olympus, Tokyo, Japan).

### Histochemistry

Rats were deeply anesthetized with sodium pentobarbital as described above for perfusion with PBS and then with 4% paraformaldehyde in PBS (pH 7.4). The brains were removed, postfixed in 4% paraformaldehyde in PBS, and frozen in crushed dry ice. Coronal sections (14 µm thickness) were cut on a cryostat and mounted on glass slides (MAS-coated glass slide; Matsunami Glass, Osaka, Japan).

For F-actin staining, the sections were incubated in phalloidin-TRITC (0.1 ng/ml; Sigma-Aldrich) overnight at 4°C before imaging on an Olympus AX-80T light microscope. For CaMKIIα immunohistochemistry, the sections were permeabilized for 15 min with 0.2% (vol/vol) Triton X-100 in PBS and then blocked with 5% normal goat serum in PBS before incubating with anti-CaMKIIα monoclonal antibody (6G9, MA1-048, 1:100; Invitrogen) overnight at 4°C followed by Alexa Fluor 488 anti-mouse IgG (1:200; Invitrogen) for 1 h. Sections incubated in a solution lacking primary antibody exhibited no specific staining. The sections were treated for 15 min with 4′,6-diamidino-2-phenylindole [(DAPI) 10236276001, 1 µg/ml; Roche Diagnostics] and then washed three times (10 min/wash) with PBS before imaging on an Olympus AX-80T light microscope or a Zeiss LSM 700 confocal microscope.

### Data analysis

Control and HFS(500) were derived from contralateral and stimulated dentate gyrus, respectively, in this study. Two contralateral tissues were excluded from analysis because of damage during sampling. The average signal intensities for *Camk2a* mRNA, F-actin, and CaMKIIα were measured with Metamorph software (Molecular Devices). Statistical analyses were performed using GraphPad Prism 6 (GraphPad Software) and Microsoft Excel with Statcel 3 (OMS, Japan). Paired continuous data from LTP experiments were assessed using paired *t­-*tests or Wilcoxon signed-rank tests. Multiple group comparisons were assessed using one-way ANOVA followed by Scheffé’s *post hoc* test when significant main effects were detected. Quantitative data are presented as the mean±standard error of the mean (s.e.m.).
